# Supplementary material for: Finger-palm synergistic soft gripper for dynamic capture via energy harvesting and dissipation
Source: Nat Commun. 2022 Dec 13;13:7700. doi: 10.1038/s41467-022-35479-9 (PMC9747793; doi:10.1038/s41467-022-35479-9)
Supplement: Supplementary file 1 — Supplementary information [file 41467_2022_35479_MOESM1_ESM.pdf]

Supplementary Information for

Finger-palm synergistic soft gripper for dynamic capture via energy harvesting and dissipation

Authors:

Yin Zhang[1], Wang Zhang[2], Pan Gao[1], Xiaoqing Zhong[1], Wei Pu[1*]

Affiliations:

1. School of Aeronautics and Astronautics, Sichuan University, Chengdu, 610065, China

2. Department of Mechanical Engineering, Massachusetts Institute of Technology, Cambridge, 02139, USA

*Corresponding author Email: Pwei@scu.edu.cn

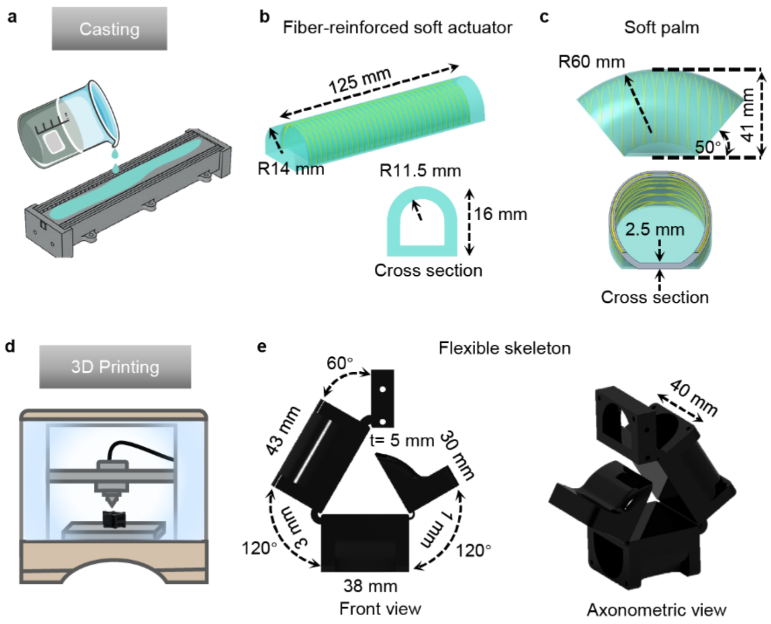

Supplementary Fig. 1. Design and fabrication of a finger and palm. a Both a fiber-reinforced bending actuator and a palm are made of silicone rubber material by casting. Their design parameters are shown in **b** and **c** respectively. **d** A flexible skeleton is made of thermoplastic polyurethane (TPU) by 3D printing technology based on Fused Deposition Modeling (FDM). **e** Front and isometric view of a flexible skeleton, including some design parameters such as joint bending angle, thickness, length, width, etc.

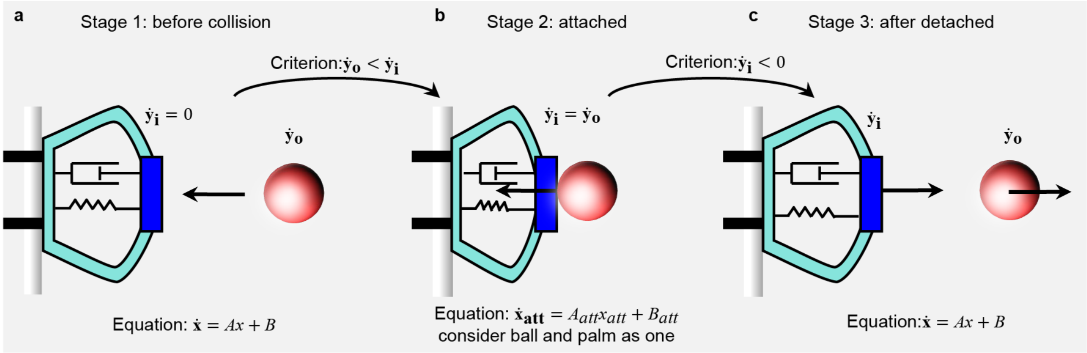

Supplementary Fig. 2. Schematic of the multi-physics model to predict the palm/ball dynamics and gas exchange. We divide the collision between the ball and the palm into three stages. **a** Before collision. The ball shoots towards the palm along a rail but has not yet touched, and the deformation of the palm at this stage is zero. **b** Attached. The ball is in complete contact with the palm and moves together, and we consider the ball and the palm as one. **c** After detached. The ball's motion is reversed

and wholly separated from the palm.

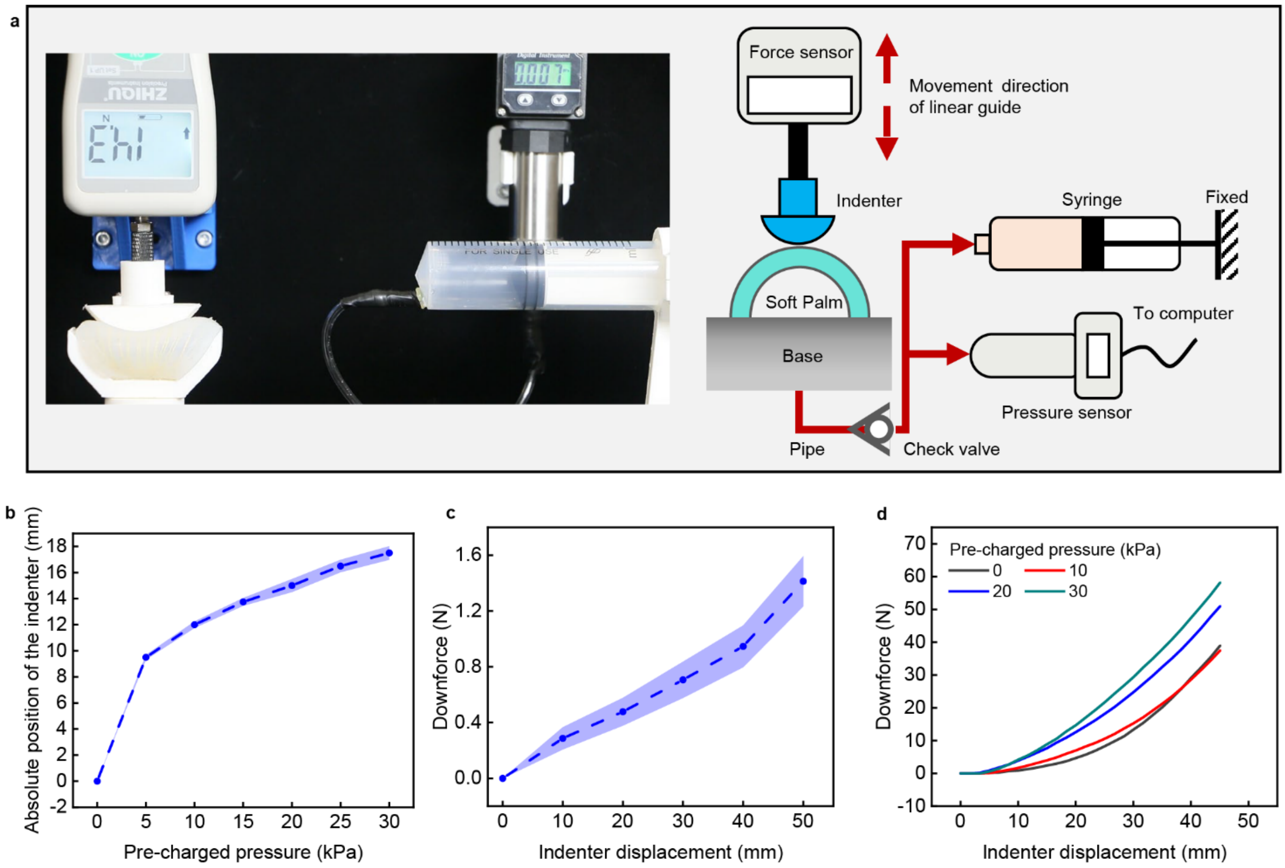

Supplementary Fig. 3. Characterization of the primary performance of the palm. a Experimental platform and its schematic diagram. It mainly includes a push-pull force sensor, a pressure sensor, a syringe, a check-way valve, a linear guide, and the palm being tested. **b** Calibrate the absolute position of the indenter. Since the palm is pre-inflated and deformed after a collision, the experimental results show the relationship between the pre-inflated pressure and the initial position of the highest point of the palm, which is used to judge when an object is in contact with the palm. **c** Stiffness of the palm itself. The pipe in the palm is connected to atmospheric pressure during the test. The results show that the palm wall has little effect on the ball's rebound due to the thin wall and low stiffness. **d** Relationship between pre-charged pressure and palm stiffness. The results demonstrate that the greater the pre-charge pressure, the greater the stiffness of the palm. However, when there is no pre-charge pressure, the deformation quickly reaches the maximum displacement.

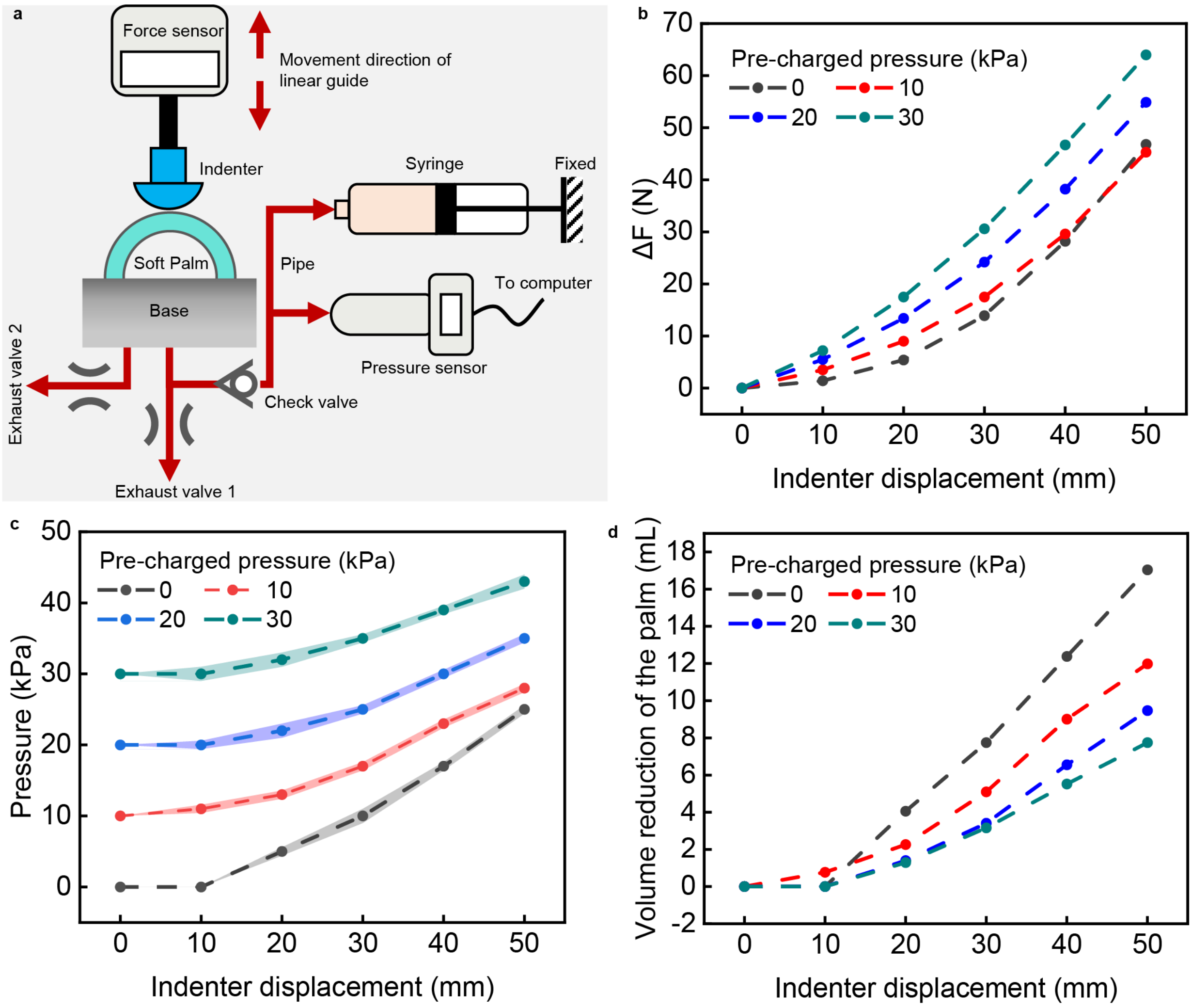

Supplementary Fig. 4. Measurement of critical parameters of the palm. a Schematic of the experimental setup. Based on the experimental setup above, an exhaust valve is added to the pipe of the palm. **b** Equivalent area of the palm. We move the indenter to a specified displacement at various pre-charge pressures and then opened exhaust valve 2, recording the maximum value of the downforce and the value on the force sensor after the pressure is released. We then use the difference ΔF to calculate the equivalent area between the indenter and the palm at each contact position. We also record the pressure on the sensor (**c**) while the indenter squeezes the palm. The palm volume reduction (**d**) is calculated from the pressure and syringe volume.

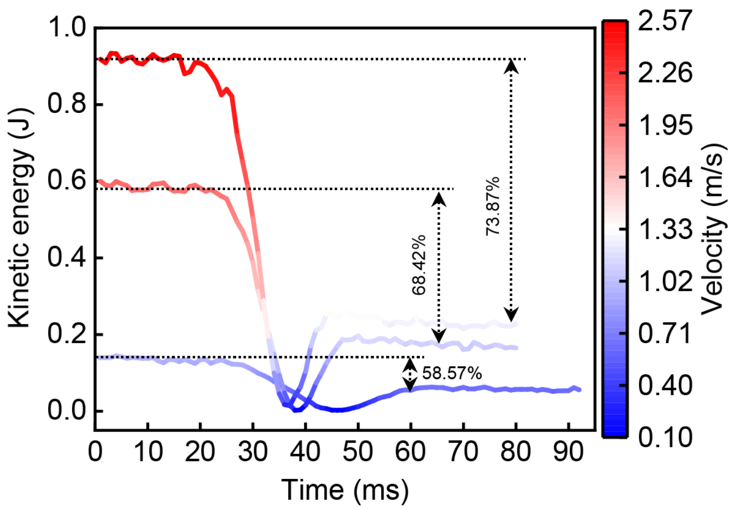

Supplementary Fig. 5. Comparison of kinetic energy attenuation laws of several collisions with different initial kinetic energy without energy harvesting.

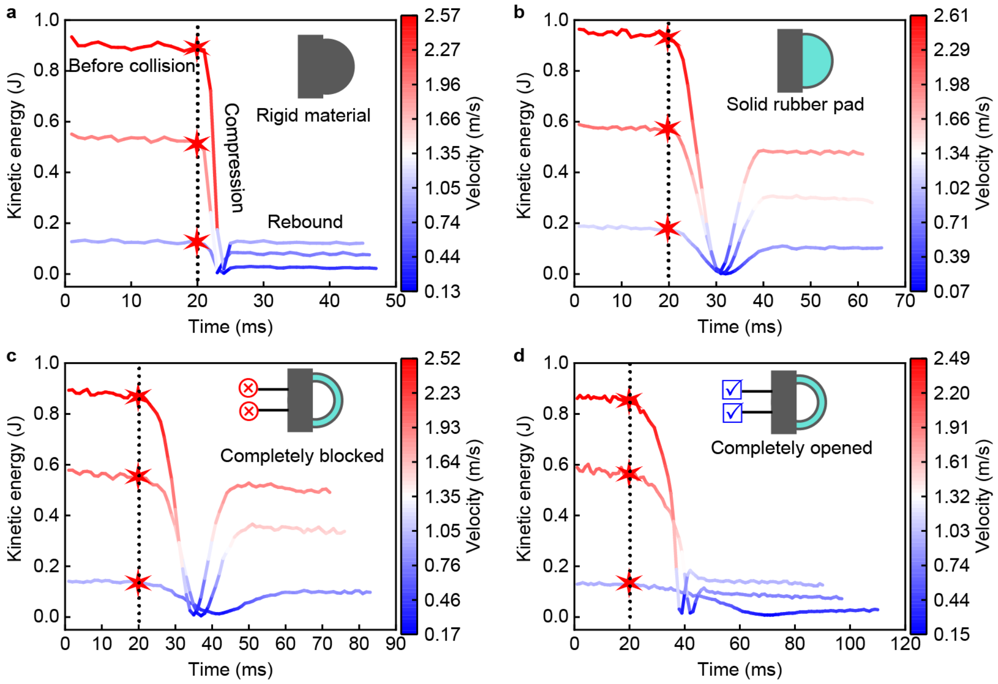

Supplementary Fig. 6. Comparison of the collision results of four typical palms. a Rigid material. The palm is made of three-dimensional (3D) printed polylactic acid (PLA). **b** Solid rubber pad. This palm is made of silicone rubber with a Shore hardness of 5 and is completely solid. **c** Completely blocked. The palm is an airbag made of silicone rubber, and its two exhaust ports are completely blocked. **d** Completely opened. The material and design are the same as (**c**), except that its two exhaust ports are completely connected to the atmosphere. Each set of graphs represents the kinetic energy dissipation of the ball during a collision, and the color map represents the velocity decay of the ball.

The ball hits the palm from 20 ms.

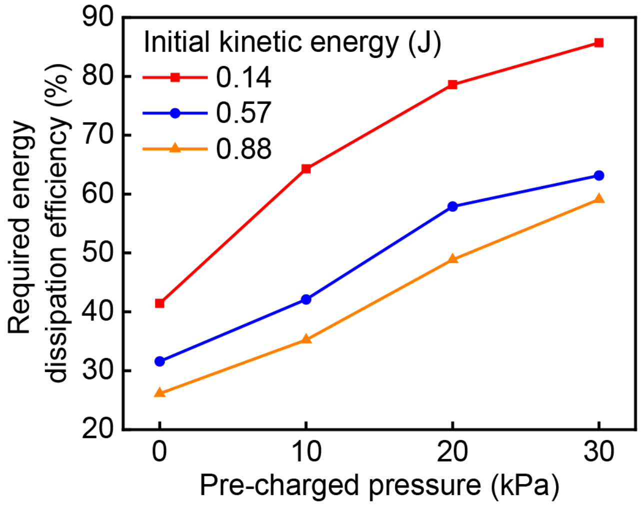

Supplementary Fig. 7. Relationship between the initial collision kinetic energy and the energy dissipation efficiency required for the FPSED mechanism under different pre-charged pressures.

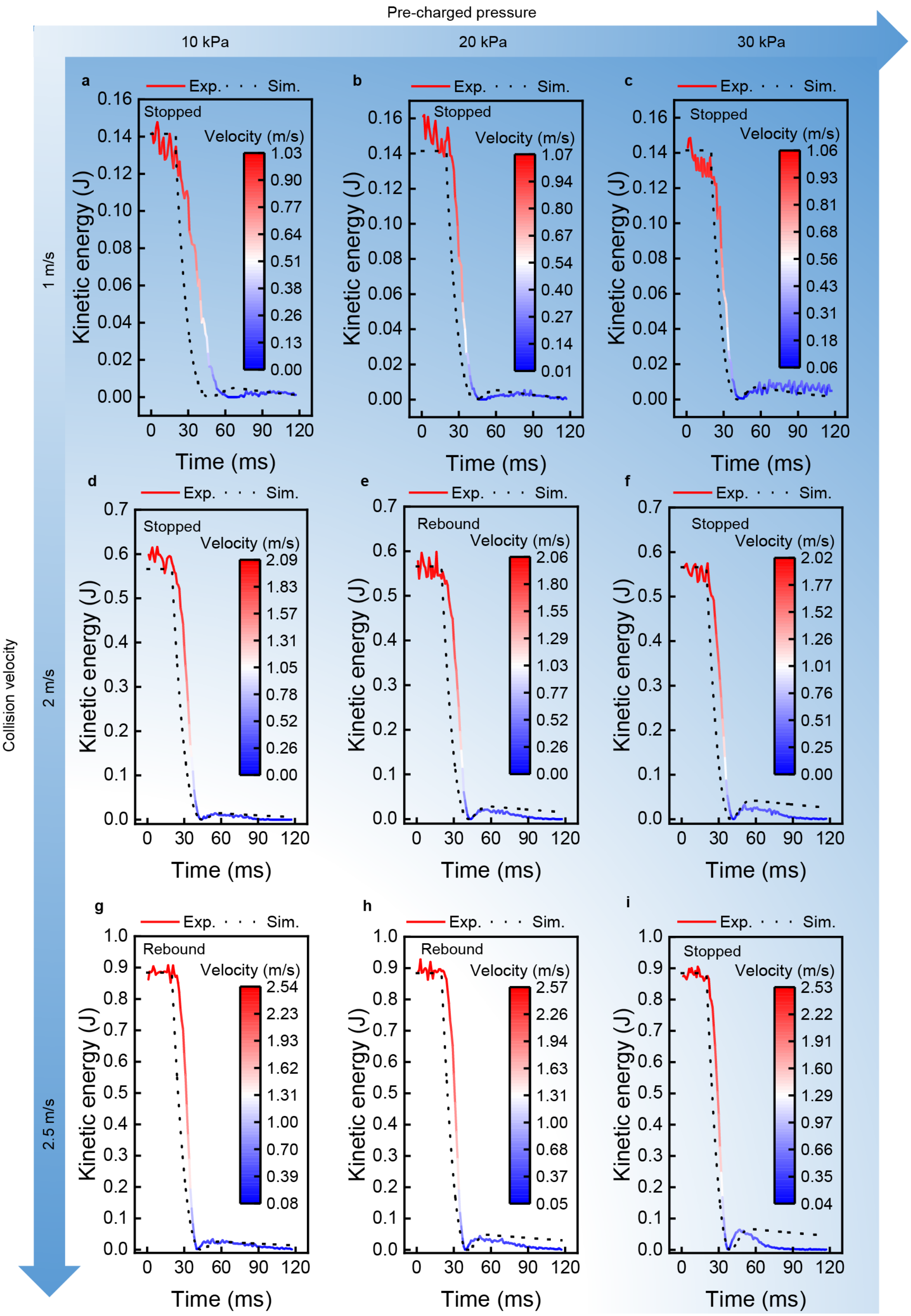

Supplementary Fig. 8. Palm compatibility for object kinetic energy. We inflate the palm from 10-30 kPa in increments of 10 kPa, respectively, and then collide the palm using a 0.283 Kg glass ball at 1 m/s, 2 m/s, and 2.5 m/s. We analyze the ball's kinetic energy dissipation efficiency and velocity attenuation and observe whether the ball bounces after collision from the recorded video. All collisions

dissipate more than 95% of the balls' kinetic energy, but in some cases (**e**, **g**, **h**), the residual energy of the balls causes them to bounce back at a very low speed. Palm's compatibility is better with low pre-charged pressure (**a**, **b**, **c**). Higher collision velocities require higher pre-charged pressure (**i**). The solid line represents experimental results (Exp.), and the dashed line represents simulation results (Sim.).

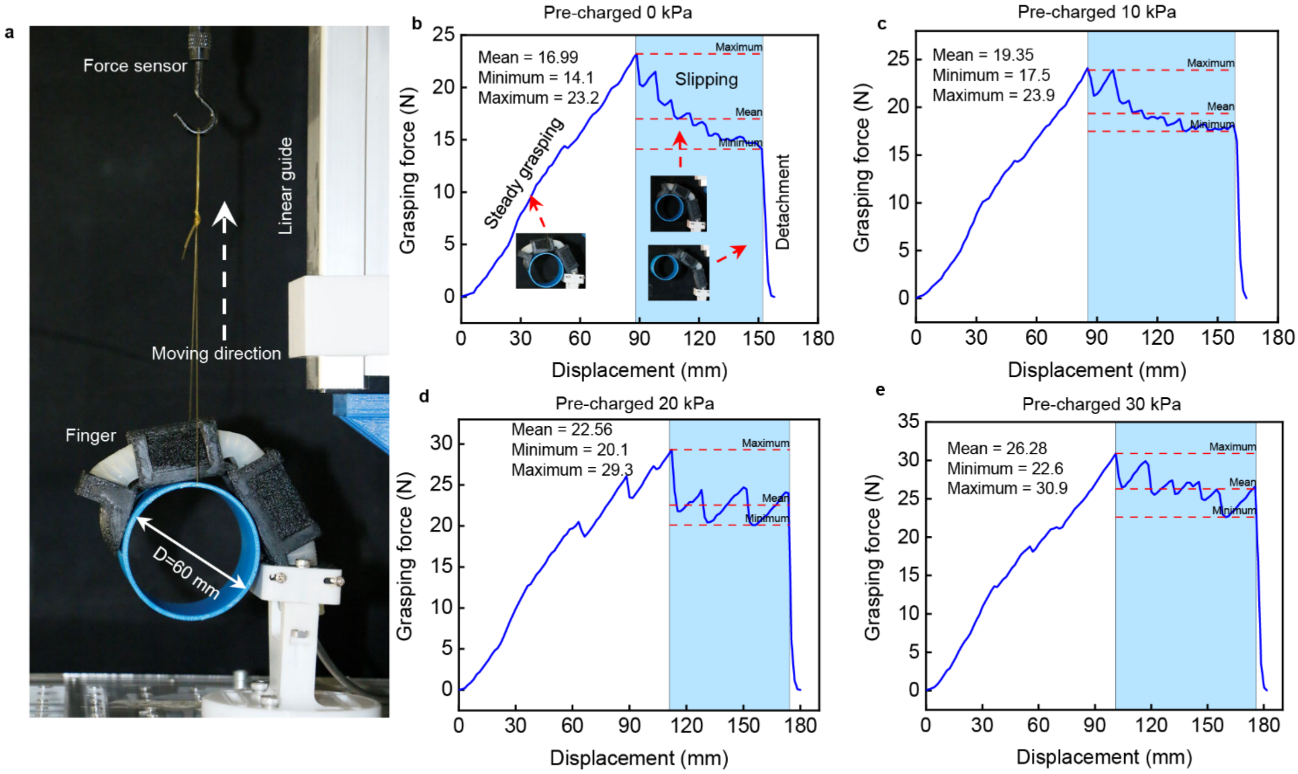

Supplementary Fig. 9. Harvested energy improves finger grip. a Experimental setup. We inflate the fingers with 0-30 kPa air pressure in 10 kPa increments and plot the grasping force. **b** When the pre-charged pressure is 0 kPa, the maximum grasping force is 23.2 N, and then with the increase of air pressure, the maximum grasping force is 23.9 N (**c**), 29.3 N (**d**), and 30.9 N (**e**). In addition, it can also be seen from the average slipping force in the slipping stage that the harvested energy significantly improves the finger grasping force, for example, from 16.99 N to 19.35 N, and finally to 26.28 N at 30 kPa with pre-charged pressure.
