# Supplementary material for: Finger-palm synergistic soft gripper for dynamic capture via energy harvesting and dissipation
Source: Nat Commun. 2022 Dec 13;13:7700. doi: 10.1038/s41467-022-35479-9 (PMC9747793; doi:10.1038/s41467-022-35479-9)
Supplement: Supplementary file 3 — Description of Additional Supplementary Files [file 41467_2022_35479_MOESM3_ESM.pdf]

Description of Additional Supplementary Files

File Name：Supplementary Movie 1

Description: Comparison of four typical palms.

File Name：Supplementary Movie 2

Description: Our proposed FPSED mechanism.

File Name：Supplementary Movie 3

Description: Our proposed FPSED mechanism with pre-charged pressure from 10 kPa to 30 kPa.

File Name：Supplementary Movie 4

Description: Two-finger gripper to capture dynamic targets.

File Name：Supplementary Movie 5

Description: Gripper integrated into a drone to capture dynamic targets.

File Name：Supplementary Movie 6

Description: Comparison of uniform motion and sequential motion.
